# Enhanced laser-driven proton acceleration using nanowire targets

**DOI:** 10.1038/s41598-020-80392-0

**Published:** 2021-01-26

**Authors:** S. Vallières, M. Salvadori, A. Permogorov, G. Cantono, K. Svendsen, Z. Chen, S. Sun, F. Consoli, E. d’Humières, C.-G. Wahlström, P. Antici

**Affiliations:** 1 INRS-EMT, 1650 blvd. Lionel-Boulet, Varennes, QC J3X 1P7 Canada; 2 CELIA, Univ. of Bordeaux, 351 Cours de la Libération, 33400 Talence, France; 3 National Agency for New Technologies, Energy and Sustainable Economic Development, Via Enrico Fermi 45, 00044 Frascati, Rome, Italy; 4 Univ. of Rome “La Sapienza”, P. Aldo Moro 5, 00185 Rome, Italy; 5 Department of Physics, Lund University, 22100 Lund, Sweden

**Keywords:** Laser-produced plasmas, Nanowires, High-field lasers, Plasma-based accelerators

## Abstract

Laser-driven proton acceleration is a growing field of interest in the high-power laser community. One of the big challenges related to the most routinely used laser-driven ion acceleration mechanism, Target-Normal Sheath Acceleration (TNSA), is to enhance the laser-to-proton energy transfer such as to maximize the proton kinetic energy and number. A way to achieve this is using nanostructured target surfaces in the laser-matter interaction. In this paper, we show that nanowire structures can increase the maximum proton energy by a factor of two, triple the proton temperature and boost the proton numbers, in a campaign performed on the ultra-high contrast 10 TW laser at the Lund Laser Center (LLC). The optimal nanowire length, generating maximum proton energies around 6 MeV, is around 1–2 $\upmu$m. This nanowire length is sufficient to form well-defined highly-absorptive NW forests and short enough to minimize the energy loss of hot electrons going through the target bulk. Results are further supported by Particle-In-Cell simulations. Systematically analyzing nanowire length, diameter and gap size, we examine the underlying physical mechanisms that are provoking the enhancement of the longitudinal accelerating electric field. The parameter scan analysis shows that optimizing the spatial gap between the nanowires leads to larger enhancement than by the nanowire diameter and length, through increased electron heating.

## Introduction

Laser-driven ion acceleration, as obtained by interaction of a high-intensity short-pulse laser with a target, is a recent field of interest for its many diverse potential applications^1–3^. Besides being a compact source, laser-accelerated protons feature a high brillance, a short bunch duration and a large energy spread. This has catalyzed their utilization in different domains that include ultra-fast radiography^4^, novel fusion schemes^5^, high energy density matter^6^, laboratory astrophysics^7^, medical applications^8–10^, novel neutron sources^11^, cultural heritage^12,13^, material science^14–16^ and using them as injectors for larger accelerators^17,18^. Most of these applications have been currently explored with the most consolidated acceleration mechanism that is obtained on typical commercially available TW laser systems, the so-called Target-Normal Sheath Acceleration (TNSA)^19^. In this acceleration scheme, hydrogen containing contaminants are accelerated at the rear surface of a thin solid foil (target), typically made of gold (Au), aluminum (Al) or copper (Cu), that is irradiated by a high-intensity ($I_0>10^{18}\,\hbox {W/cm}^2$), short pulse (< 1 ps) laser operating in the near-infrared spectral range. Since about two decades, scientists are trying to find ways to increase the proton energy and proton flux, such as to expand the portfolio of viable applications. Besides increasing the laser energy, which results in high proton energies^20^, the most straightforward method to do so is by improving the laser energy absorption on the target, a key parameter to transferring energy into the accelerated ions.

In the past, there have been many suggestions on how to improve the target in order to maximize the laser-to-target absorption. Since one of the current trends for utilizing these sources for applications is by increasing the particle flux (for example, by increasing the shot rate), any new target proposal needs to result viable in terms of being easy and cheap to manufacture, and fast and non-stringent to align. Similar to photovoltaic applications, trapping light using micro- and nano-structured surfaces is one of the most established approaches to enhance the laser energy absorption^21–23^. In laser-driven ion acceleration, many studies have proposed nanostructuring the front target surface for this purpose, demonstrating both theoretically^24–31^, and experimentally^32–42^, an improvement in the absorption mechanisms. The drawback of such structures is that any trapping is limited to a nanometric scale in all three dimensions, which might not be always ideal. Nanometric rods standing up-right on a substrate in a brush-like geometry, also called nanowire (NW) targets, have recently been suggested for TNSA: These targets have the advantage of combining micrometric trapping thickness with nanometric structures. First theoretical and numerical estimations made by Wang et al.^43^ have shown that this target type allows for an enhanced laser energy absorption. The same nanowire geometry has been demonstrated experimentally to be very effective for enhanced THz pulse generation^44^, and several other works have demonstrated enhanced X-ray emission though greater electron heating with nano-velvets^45^ and nanowires^46–48^. The high aspect ratio between length and diameter of NWs favors an increased laser absorption due to a greater effective interaction surface area with the incoming electromagnetic (EM) wave, occurring within a few laser cycles such as to maximize the interaction with the intact NW forest. This increased interaction ejects electrons from the NW boundaries mainly through Brunel-type and $\varvec{J}\times \varvec{B}$ absorption processes, which are further accelerated in the gaps between the NWs by Direct Laser Acceleration (DLA) before re-collision with the target bulk^49^, where the electrons seed a cascade of impact ionization events. This leads to a denser and hotter electron cloud at the rear side of the target and generates a more intense accelerating sheath electric field driving the TNSA mechanism. Only two experimental studies have been performed to demonstrate this effect: Firstly, Khaghani et al.^50^ irradiated micro-pillar targets with a 80 J and 500 fs laser. They obtained a maximum energy enhancement ratio of up to 2.4 and 20 times more protons in the spectrum. They show a measured hot electron temperature increase by a factor of 2 at lower laser intensity. Secondly, Dozières et al.^51^ used NW targets and obtained a maximum energy enhancement factor in the range 1.5–2. In the same work, an experimental evaluation of the influence of *d* and *g* was performed, recommending a thorough evaluation of influence of length *l* in a subsequent study. In both works, a direct comparison between nanowires and flat targets having the same thickness within a systematic approach is missing, preventing conclusions to be drawn.

In this work we present a systematic study of enhanced laser-driven proton acceleration using Cu nanowire targets with regards to the geometrical parameters (diameter, gap and length). Our findings are supported and complemented numerically by Particle-In-Cell (PIC) simulations, which enabled a full parametric investigation. The effect of the NW’s geometrical parameters are related to theoretical models for plasma expansion in vacuum^52^. The experimental study is complementary to Dozières et al.^51^ and focuses on the NW length *l* as the main investigated parameter. Using experimental NW targets, we find average proton enhancement ratios of 2 and 3 for maximum energy and temperature respectively, as well as multiple-fold proton number enhancement. We relate these improvements to an enhanced TNSA-related electric field produced by a stronger hot electron yield, both in density $n_\text {e}$ and temperature $T_\text {e}$. We find that the number of hot electrons generated by the interaction is dominantly influenced by the nanowire diameter, whereas a larger gap opening boosts the electron temperature up to a particular optimized gap size value. Between both parameters, the gap size has stronger influence compared to the nanowire diameter with regards to the laser energy absorption.

## NW geometry optimization

**Figure 1:**
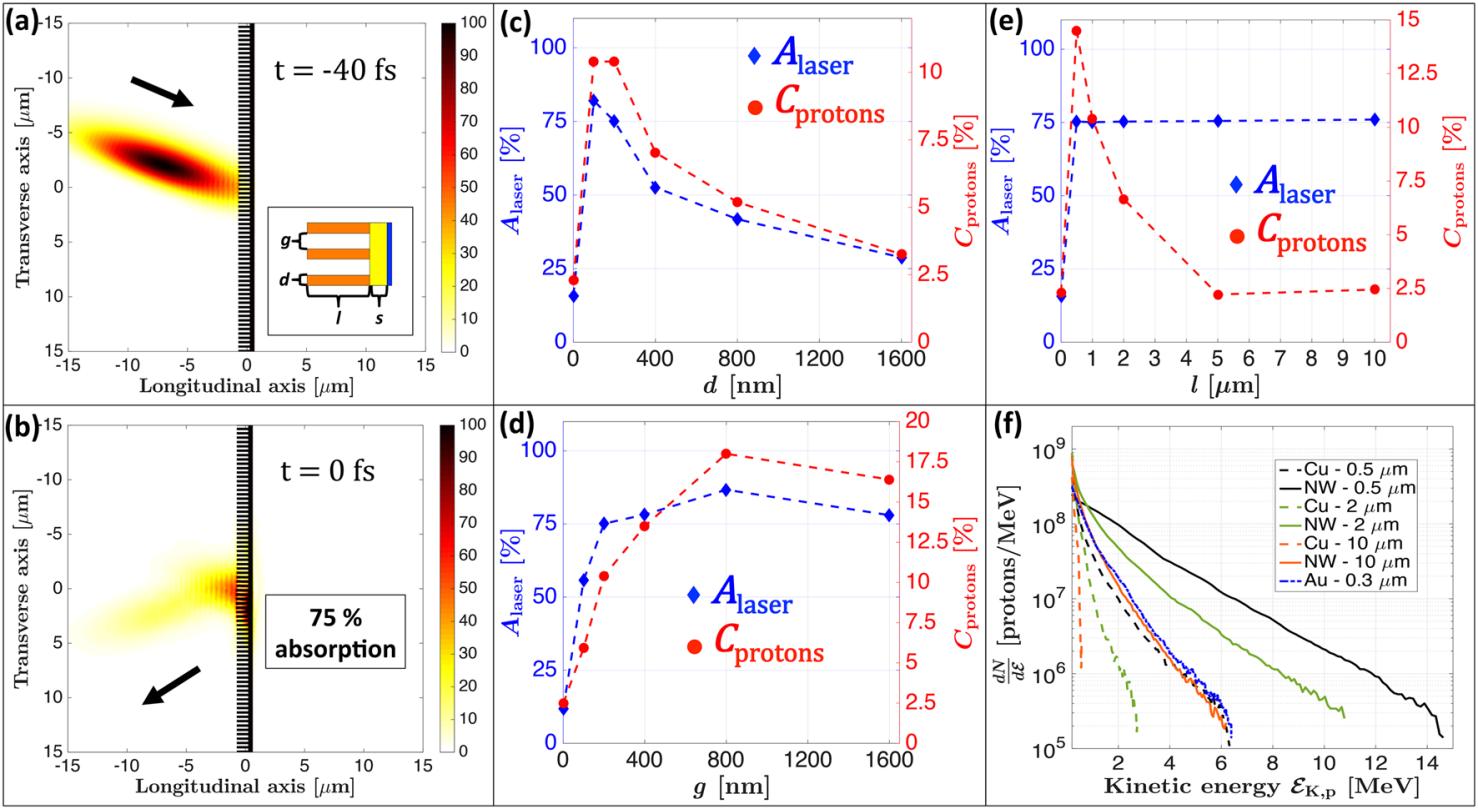
Numerical optimization of NW geometry through PIC simulations using the 2D3V PICLS code. (**a**) EM intensity and simulated target shown just before the laser interaction at $t=-40$ fs. Color scale is in percentage of maximum intensity. The inset is a NW scheme defining the geometry parameters *l*, *d* and *g*; target is composed of copper NW (orange), gold substrate (yellow) and a proton layer (blue). (**b**) EM intensity and simulated target shown during the laser-target interaction at $t=0$ fs. (**c**–**e**) Variation of laser energy absorption (blue diamonds—left scale) and laser-to-proton conversion efficiency (red points—right scale) for different NW (**c**) diameters *d*, (**d**) gaps *g* and (**e**) lengths *l*. (**f**) Simulated proton spectra for NWs with $d=g=200$ nm (same values as the experimental working point) and three different NW lengths of 0.5, 2 and 10 $\upmu$m (solid lines), along with their thickness-equivalent Cu reference foils and the bare Au substrate of 0.3 $\upmu$m (dotted lines).

We performed PIC simulations using the 2D3V PICLS code^53^ to determine the optimal NW geometry for proton acceleration and thus to orient the NW production process. The 2D PIC simulations allow to investigate the role of NW length and check the optimal parameter range for the specific experimental configuration used in this work. In addition, the extensive parametric investigation considering all three parameter (*d*, *g* and *l*) allows a more comprehensive understanding of how the increased absorption and electron generation result in enhanced proton acceleration. Details about the simulation parameters can be found in *Methods*. The simulation geometry is shown in Fig. 1a. We performed different parametric simulations to optimize the diameter *d*, gap *g* and length *l* of the NWs, starting from the best case scenario depicted by Wang et al.^43^ (i.e. $d=200$ nm, $g=200$ nm and $l = 1\,\upmu$m at central laser wavelength of $\lambda _0=800$ nm) which we define as the nominal parameters. We varied each parameter independently, keeping the other parameters fixed. We can note in Fig. 1b where we present a snapshot of the simulation using the nominal parameters, a laser energy absorption of 75% compared to about 5% for a typical flat Cu foil, as is also shown in Fig. 1c–e for the best laser absorption cases (blue markers). Figure 1c shows that the laser energy absorption and laser-to-proton conversion efficiency peak for NW diameters of $d = 200$ nm ($\lambda _0/4$). Laser absorption and conversion efficiency both increase for $d<100$ nm, as consistent with the work of Martinez et al.^54^, and then decrease after $d=200$ nm. This can be justified as follows: for small NW diameters $d<100$ nm, increasing *d* leads to higher laser energy absorption through a greater number of electrons interacting with the laser pulse, whereas for values larger than $d=200$ nm the area irradiated by the laser is closer to a flat surface as it reflects more energy from the NW tip surface. Figure 1d shows the variation of absorption with gap distance *g*, the laser energy absorption is already near-maximal at $g=200$ nm ($\lambda _0/4$)^43^, whereas the proton conversion efficiency optimum is found at $g = 800$ nm ($\lambda _0$). Interestingly, the laser absorption increases only slightly between 200 and 800 nm, while the laser-to-proton conversion efficiency increases significantly (almost doubles, passing from 10 to 18%). This difference among the two absorption mechanisms is due to the ejected electrons that are accelerated within the laser field in the gap region before re-collision with the target bulk: For too small gaps, the electrons reach the next NW with lower kinetic energy (electronic temperature) than for larger gaps. Therefore, larger gaps lead to a greater energy transfer to the protons, a phenomenon also observed and explained in the work of Blanco et al.^27^ using triangular nanostructures and found also in other works^42,54,56,57^. Concerning the NW length variation exposed in Fig. 1e, we see that the laser energy absorption (blue diamonds) improves very strongly from $l=0\,\upmu$m (flat target substrate of thickness $s=300$ nm) to $l=0.5\,\upmu$m, whereas for $l>0.5\,\upmu$m it only increases by less than 1%. Concerning the laser-to-proton conversion efficiency (red dots), it is best for the shortest NWs and decreases for longer NW lengths. This is similar to what is observed in the TNSA regime when increasing the thickness of flat targets^55^. More precisely, electrons ejected from longer NWs will go through a larger effective material thickness and thus lose more energy (i.e. lower electron temperature) before reaching the rear target surface where they establish the accelerating sheath electric field. Hence, even if longer NWs lead to a greater laser energy absorption and therefore to greater number of ejected electrons, using long lengths also increases the energy loss of electrons during their re-collision with the target (wires and substrate), which ultimately produces lower laser-to-proton conversion efficiency. This effect is exhibited in Fig. 1f where we compare the simulated proton spectra for NW lengths of $l=0.5$, 2 and 10 $\upmu$m with respect to their thickness-equivalent reference flat Cu foil spectra, along with the bare Au substrate case. All NW targets yield higher energies than their respective reference Cu foils, moreover the least performant NWs (i.e. $l = 10\,\upmu$m) provide equivalent kinetic energies than the most performant flat foil case, namely the 0.3 $\upmu$m-thick Au substrate. The estimated maximum energy enhancement ratio is about 3 for $l=0.5\,\upmu$m, 4.4 for $l=2\,\upmu$m and goes up to a factor of 10 for $l=10\,\upmu$m due to the low energies generated for such large thickness. We limited our simulations to 2D PIC simulations presented here, without exploring 3D PIC simulations, since our simulations already give a sufficiently complete idea of the main phenomena with regards to the enhanced acceleration mechanism, as well as offering a comprehensive view of the underlying phenomena occurring within the geometry optimization. However, we expect to observe lower enhancement ratios in the experiment since 2D PIC simulations are known to overestimate the electrons energies by a factor in the range of 1.5-2^58,59^ through greater $\varvec{J}\times \varvec{B}$ electron heating, although this effect can be compensated by the shorter travel time of hot electrons in the NW interspace in 2D^60^. This proton energy overestimation is further amplified for NWs due to an overestimated electron confinement, leading to stronger TNSA electric field^60^. In the experiment, described in the following section, we did not use the proton-optimized gap of 800 nm due to limitations in the fabrication methodology and this will be the subject of further investigations. Nevertheless, the experimentally used gap of $g=200$ nm already provides a substantially increased laser-to-proton conversion efficiency by a factor of 5 with respect to the flat target case, moreover being near-optimal for the laser energy absorption as shown in Fig. 1d (blue diamonds).

## Experimental campaign

**Figure 2:**
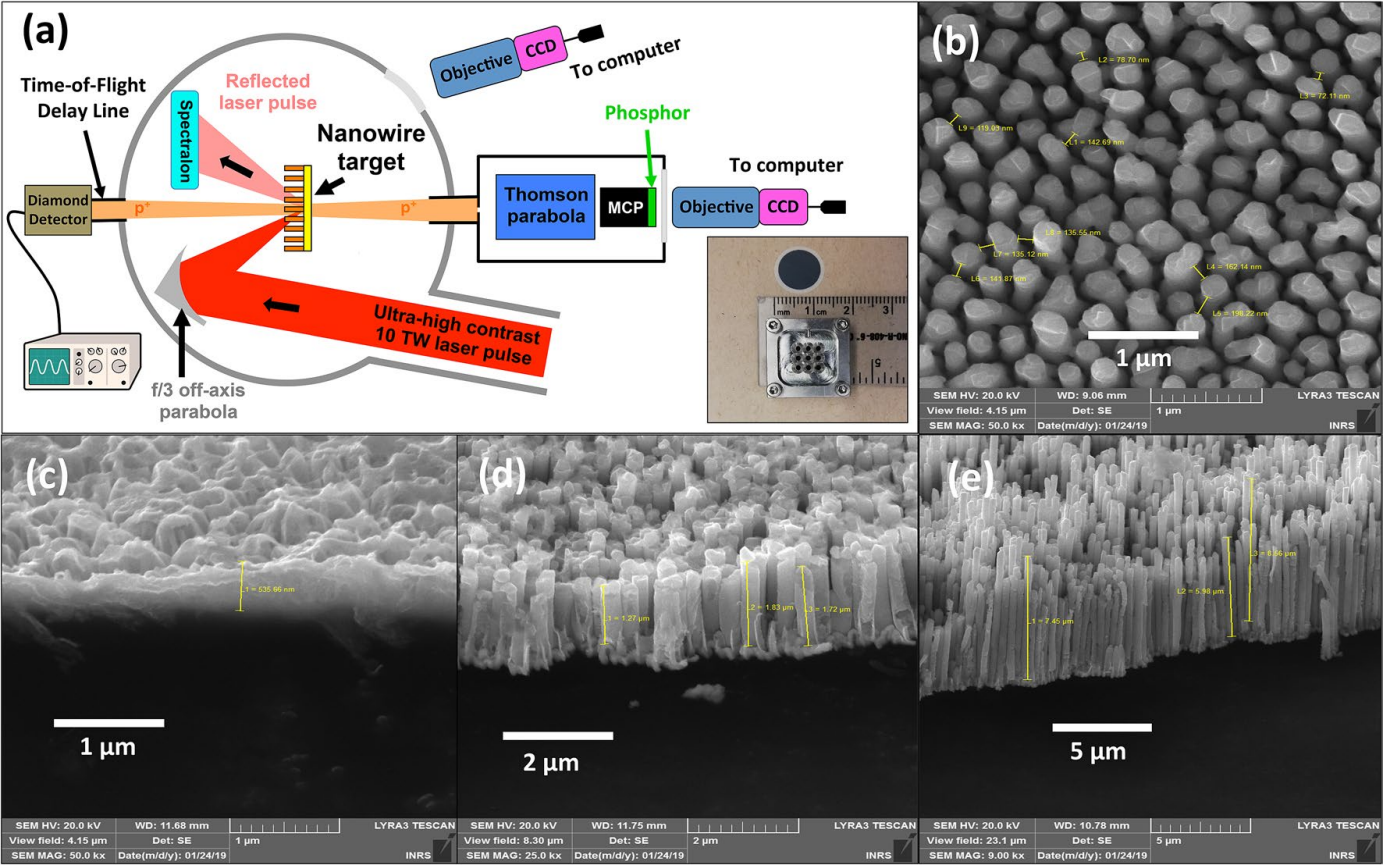
Experimental setup. (**a**) Schematic of the experimental setup showing the three main proton monitoring systems: a TP spectrometer at $0^\circ$, a TOF line at $180^\circ$ and a Spectralon diffuser. (inset) Target holder specifically designed for holding the NW discs. (**b**–**e**) SEM images showing (**b**), a top view of the nanowire target. (**c**–**e**) different NW targets of different lengths (0.3 $\upmu$m in (**c**), 1.5 $\upmu$m in (**d**) and 8 $\upmu$m in (**e**).

We performed experiments on the high-power Ti:Sapphire laser of the Lund Laser Center (LLC)^61^ in Lund (Sweden). The system benefits of a double plasma mirror (DPM) configuration providing an Amplified Spontaneous Emission (ASE) pedestal contrast better than of $10^{-11}$ at 100 ps and $10^{-9}$ at 3 ps before the main pulse, with an energy transmission efficiency of 40%. Having an ultra-high contrast is essential for this type of study in order to keep the nanostructures intact when the main pulse arrives. The laser pulses had an energy of ${\mathcal {E}}_L =0.35$ J on target, a duration of $\tau _L=35$ fs yielding a peak power of 10 TW at a central wavelength of $\lambda _0 =800$ nm, and the beam was focused down using an *f*/3 off-axis parabola to a focal spot diameter of $w_\text {FWHM}=3\,\upmu$m, providing an on-target intensity of $I_0 \sim 5\times 10^{19}\,\hbox {W/cm}^2$. The p-polarized pulses were incident on the targets at an angle of $20^\circ$ with respect to target-normal direction to allow for the measurement of the reflected laser pulse and to avoid sending the reflection back in the laser system as when using $0^\circ$ incidence, while preserving an efficient TNSA mechanism. The proton beam spectrum was monitored by a calibrated Thomson Parabola (TP) spectrometer placed at $0^\circ$, coupled to a microchannel plate-phosphor assembly for particle detection. In order to monitor particles accelerated in the laser-backward direction, we used a Time-of-Flight (TOF) delay line oriented at $180^\circ$ with respect to the TP spectrometer. In the TOF detector, the ions were detected using a Chemical Vapor Deposition (CVD) diamond detector^62–64^. The reflectivity of the target was measured using a Spectralon diffuser placed at the specular reflection angle compared to the impinging laser; an image of the scattered light was recorded on a CCD through a window of the chamber. Details of the experimental setup are presented in Fig. 2. As targets we used Cu NW targets, with an average diameter of $d = 200$ nm, gap of $g = 200$ nm and five different verified lengths of $l=0.5$, 1, 2, 5 and 10 $\upmu$m. The used NW geometry provided an areal density of $6.25\,\hbox {NW}/\upmu \hbox {m}^2$ (i.e. reduction of the effective electron density by a factor of 0.4 compared to Cu flat targets) and therefore approximately 44 NWs were located within the focal spot area. This NW density is close to the numerical optimum of the present study ($d_\text {opt}=200$ nm and $g_\text {opt}=800$ nm), that yields 7 NWs per focal spot, and slightly higher that the optimum reported in the work of Dozières et al.^51^ (around 1 NW per focal spot area). We expect that this difference is due to the higher laser intensity ($1.5\times 10^{21}\,\hbox {W/cm}^2$) used in their study, as increasing the intensity would require longer traveling time for attaining maximal heating of the ejected electrons, suggesting the use of larger gap distances (i.e. lower number of NW per focal spot). The NWs were grown on a $s=300$ nm thick Au substrate (the total thickness of the target being $s+l$) by electrodeposition using Anodic Aluminum Oxide (AAO) templates of 1 cm in diameter (available commercially, WHATMAN Anodisc), following the methodology described in Mondal et al.^44^ and adapted from Gao et al.^65^. The NW production methodology is easily implementable in-house and inexpensive, allowing for large amounts of targets to be produced at once. However, the method still presents some limitations to vary the parameters *d* and *g*, another reason why we focused our attention on the experimental investigation of the parameter *l*. A specific target holder was designed for the NW discs allowing for 9 repetitive shots per disc, as shown in the inset of Fig. 2a. In order to test the repeatability of the data, and ensure statistically valid information, each target type was irradiated several times (3-18 times, depending on the available targets). Scanning Electron Microscope (SEM) images of the NW targets are shown in Fig. 2b–e for three different NW lengths. One can see that for very short NWs (NW length of 0.3 $\upmu$m as indicated in Fig. 2c), the target surface appears to be very irregular and rugged, due to the too short growth time. Longer growth times form the as-expected forests of wires, as visible in Fig. 2d (1.5 $\upmu$m) and 2e (8 $\upmu$m). The NW lengths *l* are measured using the postprocessing software of the SEM microscope, so that the NW lengths *l* are calibrated with the chemical reaction growth time. Reference shots were also taken on five types of Cu foils with thicknesses equivalent to the different NW lengths *l* (0.5, 1, 2, 5 and 10 $\upmu$m), along with the NW bare Au substrate.

**Figure 3:**
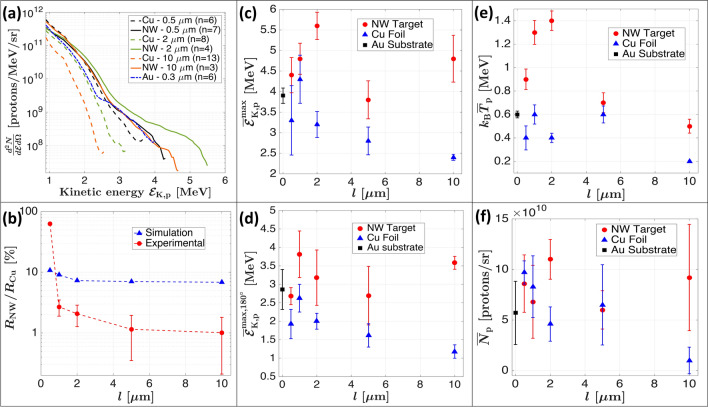
Experimental results for NW length investigation. (**a**) Averaged spectra for NW targets (solid lines) along with their thickness-equivalent Cu reference foils and the bare Au substrate of 0.3 $\upmu$m (dotted lines). (**b**) Reflectivity ratios of NW targets and Cu foils as obtained experimentally from the Spectralon diffuser (red points) and through PIC simulations (blue diamonds). Proton spectra characteristics (**c**–**f**) shown for NW targets (red circles), Cu reference foils (blue diamonds) and the bare 300  m-thick Au substrate (black squares). (**c**) Average maximum proton energy in the forward acceleration direction $\overline{{\mathcal {E}}}_\text {K,p}^{\text {max}}$. (**d**) Average maximum proton energy in the backward acceleration direction $\overline{\mathcal {E}}_\text {K,p}^{\text {max}, 180^\circ }$. (**e**) Average proton temperature $k_B{\overline{T}}_\text {p}$ as obtained by a linear fit on the log-plotted spectra in the high energy section. (**f**) Average integrated proton numbers ${\overline{N}}_\text {p}$ for ${\mathcal {E}}_\text {K,p}>1$ MeV. The cumulated number of shots are of 7, 7, 4, 4 and 3 for NWs of length $l=0.5$, 1, 2, 5 and 10 $\upmu$m respectively. For Cu foils, the cumulated number of shots are of 6, 18, 8, 10 and 13 for thicknesses of 0.5, 1, 2, 5 and 10 $\upmu$m respectively, as well as 6 shots the Au substrate. The shown uncertainties are the total standard deviations (instrument accuracy and shot-to-shot fluctuation summed in quadrature).

**Figure 4:**
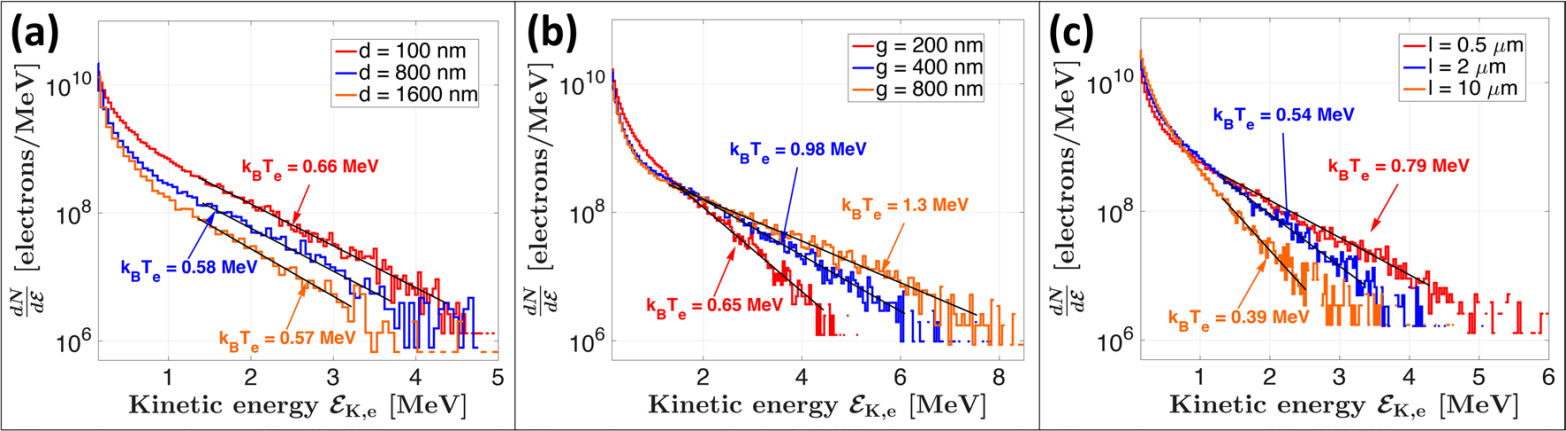
Simulated electron spectra at t = 40 fs after the interaction with the laser pulse for different NW (**a**) diameters *d*, (**b**) gaps *g* and (**c**) lengths *l*. Values of *d*, *g* and *l* are chosen for better visualization. The black lines correspond to linear fits in the relevant energy range for the retrieval of the hot electron temperature $T_\text {e}^\text {hot}$.

Figure 3a shows the averaged proton spectra for different target types. Only three NW lengths ($l=0.5$, 2 and 10 $\upmu$m) are shown for better visualization of the data, along with their respective reference Cu foils. Considering the reference Cu foils, one can observe an improvement in the proton yield and maximum proton energy with decreasing foil thickness, the expected behavior for the TNSA mechanism. We find that any NW target we used results in an equivalent or higher proton yield and maximum proton energy than what is obtained with the flat targets, including the bare Au substrate, in a very similar situation to what is observed through simulations (see Fig. 1f). The NW length that yields the highest proton energies is found to be $l=2\,\upmu$m with maximal proton energy of 5.6 MeV compared to 3.2 MeV for a Cu foil of equivalent thickness, giving a mean enhancement ratio of 1.8. Indeed, for NW lengths below 2 $\upmu$m, the wires are not yet formed as well-defined cylinders (see Fig. 2c). This decreases the laser energy absorption and therefore reduces their performance in enhancing the TNSA acceleration mechanism. Nevertheless, a non-negligible enhancement is observed even for the rough surfaces as for the case of $l = 0.5\,\upmu$m, and is likely to be attributed to a different absorption mechanism such as from stochastic incidence angles as shown in Cerchez et al.^66^. As a comparison, the mean enhancement ratio for maximum energy with $l=10\,\upmu$m is of about 2, however, the absolute maximum energy is slightly lower, 4.8 MeV, as expected for thicker targets. Hence, there is a compromise when choosing the working point: Short NW lengths produce higher proton energies, but are difficult to manufacture in a well-defined NW geometry. Longer NW lengths produce well-defined NW forests yielding high laser energy absorption, but decrease the TNSA mechanism efficiency due to the increased thickness^55^. In Fig. 3b we show the experimental and simulated laser-energy reflectivity ratios between NW targets and reference Cu foils. We can note that the reflectivity ratio obtained from PIC simulations very slowly increases from 7% for a target thickness of $l=10\,\upmu$m to 10% for a thickness of $l=0.5\,\upmu$m. The experimental reflectivity heavily increases for thicknesses below 2 $\upmu$m since NWs become too short and form the aforementioned very rough surface (see Fig. 2c), which explains the less performant NWs with lengths of $l= 0.5$ and 1 $\upmu$m. Nevertheless, we observe a good agreement between the simulated and experimental reflectivity ratio in terms of functional trend and amplitude, moreover also exhibiting the inverse trend as observed in Fig. 1e for laser energy absorption. The difference is due to the fact that during the experiment only the energy reflected in the specular direction is captured by the CCD looking at the Spectralon. Part of the energy is dispersed also by non-aligned nanowires, which leads to an underestimation of the total reflectivity. Figure 3c–d show the maximum proton energy measured on the TP spectrometer placed at $0^\circ$ in the forward acceleration direction $\overline{\mathcal {E}}_\text {K,p}^\text {max}$, in addition to those recorded at $180^\circ$ in the backward acceleration direction $\overline{\mathcal {E}}_\text {K,p}^{\text {max},180^\circ }$ using the TOF lines equipped with a diamond detector. One can see that all the NW types are univocally superior in maximum energy compared to their equivalent Cu foils and also with respect to the bare Au substrate. TP measurements depicted in Fig. 3c show that the highest maximum energy is achieved for a NW length of 2 $\upmu$m, before decreasing with shorter NWs of 0.5 and 1 $\upmu$m. This represents a different but complementary trend than what is shown with simulations on Fig. 1e, but is explained by the fact that experimental NWs with thickness below 2 $\upmu$m do not form a uniform and well-defined NW forest compared to longer lengths. Consequently, this effect decreases the laser energy absorption and thus also the efficiency of the TNSA mechanism. As a results, a decrease in performance is observed compared to a more efficient TNSA mechanism for shorter NWs, hence why the optimum is slightly shifted at $l=1\,\upmu$m in backward direction. This suggests that the experimental optimal length is in the range of *l* = 1–2 $\upmu$m. Some manufacturing problems occured with the $l=5\,\upmu$m target, preventing to obtain perfectly flat NW targets. As a result, the performance of $l=5\,\upmu$m is lower than for $l=10\,\upmu$m. It is likely that this changed the target-normal direction and thus reduced the measured maximum energy. TOF measurements (see Fig. 2d) show that even in the backward direction the enhancement in the maximum proton energy is significant for long NWs, despite a lower absolute energy value. The correlation between forward and backward direction acceleration mechanism has already been investigated for thin foil targets, and for high contrast lasers the two target sides showed similar maximum energy trends^67–69^, with maximum energy slightly in favor of the forward acceleration scheme. The maximum proton energy in the backward direction is increased by a factor of 1.4 for target thicknesses of $l=0.5\,\upmu$m, up to a factor of 2.9 at $l=10\,\upmu$m. This effect is expected as the increase in laser energy absorption from NW geometry produces a larger amount of electrons and with higher temperatures in the plasma located also at the front target surface (the NW side), which expands and induces a charge separation that catalyzes the backward acceleration of ions. Even in the presence of nanostructures, the plasma expansion does uniformize the sheath electric field over time and produces an enhanced proton acceleration in the backward direction. On the temporal scale, the sheath electric field may have a lower peak value and gradient due to the rough NW surface compared to a flat surface, nevertheless the higher number of hotter electrons ultimately leads to a greater energy transfer to the ions also on the front target surface for backward acceleration. This is coherent with what is observed in the works of Dalui et al.^38^, Cristoforretti et al.^39^ and as well in Bagchi et al.^37^ in the sub-relativistic regime where the laser intensity ($10^{16}\,\hbox {W/cm}^2$) is sufficient to strongly ionize the target bulk and induces a charge separation that leads to the backward ion acceleration. In Fig. 3e we show the extracted energetic proton temperature $k_\text {B}{\overline{T}}_{\text {p}}$ as obtained by fitting a straight line in the high energy part of the log-spectrum just before the cutoff (i.e. Maxwell-Boltzmann distribution). The straight lines for obtaining the proton temperature are not shown in Fig. 3e for better visualization, but examples are shown in Fig. 4. The trend regarding proton temperatures is more clear than for the maximum energies; Since the temperature is an average metric over the hot proton population, it is less dependent on variations of the target-normal direction compared to the measurement to the TP spectrometer. It is clearly possible to see in Fig. 3e, the increasing proton temperature with decreasing NW length, having its optimum at $l=2\,\upmu$m, before decreasing back for $l<2\,\upmu$m due to misformed NWs. The proton temperatures are also all superior compared to their respective reference Cu foils, the enhancement ratio going up to a factor of 3.5 for $l=2\,\upmu$m. In Fig. 3f we show the total proton number per unit solid angle ${\overline{N}}_\text {p}$ obtained by integrating the spectra for proton energies $>1$ MeV. The trend here is less clear; proton numbers are slightly lower for some NW cases, but this is strongly dependent on the low energy threshold that we fixed when computing the integrated number and on the orientation of target-normal which produces a high statistical fluctuation in the measurement, given the small acceptance angle of the TP diagnostic. Nevertheless, the proton number enhancement is clear for $l=2\,\upmu$m and $l=10\,\upmu$m for enhancement ratios going up to 2.5 and 9, respectively.

As can be seen from these results, in all cases NW configurations improve the acceleration mechanism compared to flat foils of equivalent thickness, even when compared to the very thin Au substrate of 300 nm thick. This demonstrates that the enhancement mostly comes from the laser energy confinement determined by the NW parameters *d* and *g*, and is influenced to a lesser extent by the NW length *l*. The results obtained in this work are in agreement with the work of Khaghani et al.^50^ with regards to maximum energy and number enhancement ratios. Moreover, they measured hot electron temperature improvements of a factor of 2 at a lower laser intensity ($5\times 10^{17}\,\hbox {W/cm}^2$), in agreement with our factor of 2, as shown for the electron temperature calculations through simulations presented in the next section. Concerning the work of Dozières et al.^51^, the maximum energy enhancement ratios are again in good agreement with those of the present study.

## Underlying physical phenomena and discussion

In light of the experiments, we analyzed the simulations to highlight the underlying physical mechanisms responsible for the enhanced proton acceleration. In Fig. 4 we show the electron spectra immediately after the interaction of the laser pulse with the target at t = 40 fs. The hot electron temperature $T_\text {e}^\text {hot}$ was calculated by obtaining the slope of a straight line fit in the high-energy part of the log-spectra, as for a Maxwell-Boltzmann distribution where $\frac{dN}{d{\mathcal {E}}} \sim e^{-{\mathcal {E}}_\text {K}/k_\text {B}T}$. The number of hot electrons $N_\text {e}^\text {hot}$ was then calculated by integrating the spectra for energies above the ponderomotive potential ${\mathcal {E}}_\text {pond} = m_\text {e}c^2 \left( \sqrt{1+\frac{a_0^2}{2}} - 1 \right) =1.47\,\text { MeV}$, where $a_0$ is the normalized amplitude of the vector potential. On the first hand, we note from Fig. 4a that the hot electron temperature does not vary significantly with increasing NW diameter *d* (i.e. the slope does not change), however the number of hot electrons continuously decreases with increasing *d* (i.e. spectra are shifted downwards). Since large NW diameters tend towards the flat target case, this suggests that the NW diameter optimum for protons is due to an increased production of hot electrons, balanced by an increased reflection of the energy from the NW tips. On the other hand, varying the NW gap induces significant changes in the slope at high energies (i.e. the hot electron temperature) as we can observe in Fig. 4b, which is also observed in the work of Blanco et al.^27^ and Vallières et al.^42^. The empty space between the nanostructures allows to eject and heat electrons from the NW boundaries by the laser pulse through Brunel-type and $\varvec{J}\times \varvec{B}$ absorptions. These electrons are then further accelerated by DLA due to a greater time of flight before re-collision with the target bulk, thus increasing the temperature of the population. Finally in Fig. 4c, we note that increasing the NW length produces a reduction of both the hot electron number, since less low energy electrons can cross an increasingly thicker target, and the hot electron temperature. This results in an increased energy loss for thicker targets.

**Figure 5:**
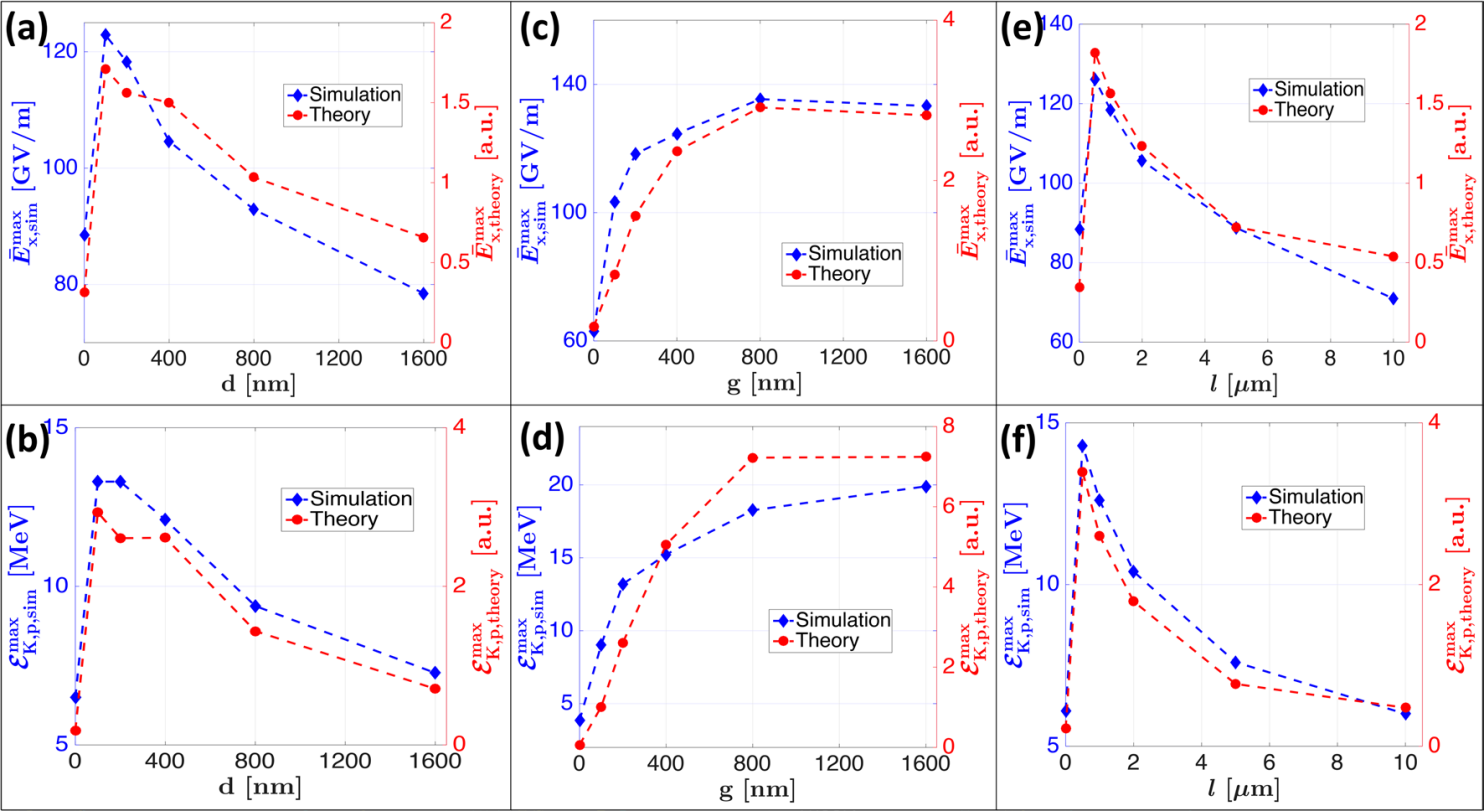
Time-averaged maximum longitudinal accelerating electric field $\overline{\varvec{E}}_\text {x, sim}^\text {max}$ (blue diamonds) calculated using (1) variation with NW (**a**) diameter *d*, (**c**) gap *g* and (**e**) length *l*, compared to $\overline{\varvec{E}}_\text {x, theory}^\text {max}$ (red dots). Simulated maximum proton energies ${\mathcal {E}}_\text {K,p,sim}^\text {max}$ variation with NW (**b**) diameter *d*, (**d**) gap *g* and (**f**) length *l*, compared to ${\mathcal {E}}_\text {K,p,theory}^\text {max}$ (red dots) calculated using (2).

Showing that the behavior of $n_\text {e}$ and $T_\text {e}$ with nanostructured targets still respects fundamental equations of the TNSA mechanism, as presented by the work Mora^52^, denotes a pure enhancement of the hot electron cloud by the nanostructures. To show that this occurs without any new unexpected effects facilitates the comprehension of the enhancement process. This is very important in order to guide the subsequent advances in enhanced laser-driven proton beams with nanostructures. To do so, we have checked the correspondance of $N_\text {e}^\text {hot}$ and $T_\text {e}^\text {hot}$ with $E_\text {sheath} = \sqrt{n_\text {e} k_\text {B} T_\text {e}/\varepsilon _0}$ and have first calculated $\overline{\varvec{E}}_\text {x,theory}^\text {max} \propto \sqrt{N_\text {e}^\text {hot}k_\text {B}T_\text {e}^\text {hot}}$, and further evaluated the maximum longitudinal electric field from the simulations $\overline{\varvec{E}}_\text {x,sim}^\text {max}$. More precisely, $\overline{\varvec{E}}_\text {x,sim}^\text {max}$ is investigated in the simulations as the time-averaged maximum longitudinal electric field defined as follows:

1 $$\begin{aligned} \overline{\varvec{E}}_\text {x,sim}^\text {max} = \frac{1}{\tau _\text {acc}} \int \limits _{t_0}^{t_f} \text {max}_\text {x,y} \Big [ \varvec{E}_\text {x}\text {(x,y,t)} \Big ] \mathrm {d}t \end{aligned}$$

where $t_0$ is the laser pulse interaction time with the target, $t_\text {f}$ is the time where the simulation ends and $\tau _\text {acc} = t_\text {f} - t_0$ is the acceleration time. The metric presented in Eq. (1) allows to remove the temporal variation of the electric field, which is disturbed compared to the typical flat target case due to the NW shape, and rather looks at the global effect as is observed with the simulated or measured proton spectra. The variation of $\overline{\varvec{E}}_\text {x,sim}^\text {max}$ with *d*, *g* and *l*, compared to $\overline{\varvec{E}}_\text {x,theory}^\text {max}$, is shown in Fig. 5. As it is possible to observe in Fig. 5a–e, there is a clear proportionality between $\overline{\varvec{E}}_\text {x,sim}^\text {max}$ and $\overline{\varvec{E}}_\text {x,theory}^\text {max}$ as they exhibit the same functional trend. Moreover, all parameters show the same trend and optima ($d_\text {opt}$ = 100-200 nm, $g_\text {opt}$ = 800 nm and $l_\text {opt}=0.5\,\upmu$m) as for the geometry optimization presented on Fig. 1. This brings a very comprehensive view of the effect of *d*, *g* and *l* on the TNSA mechanism. In particular, *d* increases the hot electron density, *g* is the driver for hot electron temperatures, which combined together produce an enhanced accelerating sheath electric field that ultimately leads to improved proton beam characteristics. This latter parameter *g* is key to achieve the highest value of temperatures and thus of the rooted product $\sqrt{N_\text {e}^\text {hot}k_\text {B}T_\text {e}^\text {hot}}$ for *g* = 800 nm, in agreement with the proton optimum presented in Fig. 1d. We have further verified the correspondence with the theoretical maximum proton energy expected from Mora^52^, i.e. ${\mathcal {E}}_\text {K,p}^\text {max} = 2k_\text {B}T_\text {e} \left[ \ln \left( t_\text {p} + \sqrt{t_\text {p}^2 + 1} \right) \right] ^2$ with $t_\text {p} = \omega _\text {p,i}t/\sqrt{2\text {e}}$ being the normalized acceleration time and $\omega _\text {p,i} = \sqrt{Ze^2n_\text {e}/m_\text {i}\varepsilon _0}$ being the ion plasma frequency. Using the maximum proton energies extracted from simulations, as well the extracted hot electrons numbers $N_\text {e}^\text {hot}$ and temperatures $k_\text {B}T_\text {e}^\text {hot}$, we compare in Fig. 5b–f the theoretical ${\mathcal {E}}_\text {K,p,theory}^\text {max}$ and simulated ${\mathcal {E}}_\text {K,p,sim}^\text {max}$ maximum energies using the proportionality presented in the following Eq. (2):

2 $$\begin{aligned} \varvec{{\mathcal {E}}}_\text {K,p,theory}^\text {max} \propto 2 k_\text {B} T_\text {e}^\text {hot} \left[ \ln \left( \sqrt{N_\text {e}^\text {hot}} + \sqrt{N_\text {e}^\text {hot} + 1} \right) \right] ^2 \end{aligned}$$

As is possible to note from Fig. 5c–d, the functional behavior is well reproduced from theory (red markers in Fig. 5b–f) and is furthermore in agreement with the shapes of the laser-to-proton conversion efficiencies $C_\text {proton}$ shown in Fig. 1c, d (red markers). The linear dependence of ${\mathcal {E}}_\text {K,p}^\text {max}$ on $T_\text {e}$ and logarithmic dependence on $n_\text {e}$, combined with the previously demonstrated relationships $T_\text {e}(d,g) \simeq T_\text {e}(g)$ and $n_\text {e}(d,g) \simeq n_\text {e}(d)$ (for a fixed optimized length $l_\text {opt}$) extracted from Fig. 4, highlights the predominant importance of the parameter *g* to achieve the highest proton energies.

Regarding the discrepancy between the experimental and numerical maximum energy enhancement ratios of respectively 2 and 3, this is partially due to the very uniform and quasi-periodic geometry within the simulation box, whereas with the experimental NWs there is a distribution of gap distances within the focal spot area of the laser which lowers its efficiency. Concerning the optimal NW length *l*, shorter NWs are the most efficient to enhance the proton acceleration as correctly shown by simulations in the present study (Fig. 1e, f), down to the theoretical minimum proposed by Wang at 0.5 $\upmu$m for a 800 nm laser wavelength. However, it is experimentally challenging to manufacture well-defined forests of short NWs using the electrodeposition methodology. This reduces the performance of too short NWs and produces an experimental optimum around *l* = 1–2 $\upmu$m for this present study. On the one hand, even if long NWs are prone to generate more hot electrons due to an increased laser energy absorption (i.e. high hot electron density $n_\text {e}$), too long NWs produce an important energy loss of hot electrons going through the target bulk (i.e. lower hot electron temperature $T_\text {e}$) which has a greater impact on the maximum energy, as predicted by the Mora model. On the other hand, too short NWs, fabricated using the electrodeposition method used in this study, do not favor a high laser energy absorption due to a misformed NW forest, explaining the presence of an optimum that is clearly visible experimentally on Fig. 3e, but is not observable numerically on Figs. 1e and 5e, f. Since it is the rooted product of $n_\text {e}$ and $T_\text {e}$ that is of crucial importance for the TNSA sheath electric field enhancement, a greater number of hot electrons does not necessarily produce a more intense accelerating field if the electron temperature is consequently lower, hence emphasizing the need to find the experimental optimum as in the case of this study. Moreover, regarding the substrate thickness, it is important to note that a too thin substrate may also lead to an over-fragile structure, a substrate thickness of about $s=1\,\upmu$m or of comparable size to the length *l* is recommended.

This type of analysis is translatable and in agreement with other types of monolayered quasi-periodic nanostructures such as nanospheres in Vallières et al.^42^ or triangles in the work of Blanco et al.^27^. The best NW parameters for *d* and *g* are a compromise between high laser confinement in the interspace of NWs and reflection of the laser energy from the NW tips. According to our simulations, an optimized NW diameter *d* favors the generation of higher hot electron densities $n_\text {e}$ due to the multiple reflections of the pulse which ejects more electrons, whereas an optimized gap distance *g* boosts the electron temperature $T_\text {e}$ since electrons are accelerated by the EM wave in the nanostructure interspace before collision with the substrate, in agreement with previous numerical studies^27,42,54,56,57^. It is the proper combination of these two parameters *d* and *g* that enhances the maximal TNSA sheath electric field as described in the formula $E_\text {sheath} = \sqrt{n_\text {e} k_\text {B} T_\text {e}/\varepsilon _0}$. Regarding the forward-accelerated proton beam enhancement also obtained at a sub-relativistic intensity ($2\times 10^{17}$ W/cm$^2$) in Khaghani et al.^50^ (backward acceleration enhancement with nanostructures at sub-relativistic intensity was demonstrated in other works^36,37^), we conclude that, if the hot electron production is not hindered by too thick substrate or NW length, then the hot electron cloud reaching the rear-side of the target can be substantially boosted in terms of numbers and temperature due to the strong heating in the wire interspace even if the absorption mechanisms vary. The DLA occurring within a few laser cycle during this time of flight in the NW gap space is therefore essential and reduces the need of very strict relativistic intensities for forward ion acceleration.

In conclusion, this work is the first to present a systematic study of the enhancement provided by nanowire targets for laser-driven proton acceleration in terms of the relevant geometrical parameters *d*, *g* and *l*, but also regarding their influence on the proton spectra characteristics (proton maximum energy, temperature and total number) in the TNSA regime, hence providing a comprehensive understanding of the proton beam enhancement with nanostructured targets. Experimental evidence exhibits high enhancement ratios for the proton spectra characteristics, in agreement with PIC simulations. A geometry optimization was also performed through PIC simulations allowing to define the best parameters for the LLC laser characteristics. A larger gap value of *g* = 800 nm between the nanowires is expected to provide even higher enhancement ratios according to our simulations. The aforementioned improvements will be the subject of a subsequent study. The easy production method along with the high enhancement ratios provided by NW targets open very promising avenues for laser-driven proton beam generation on ultra-high power laser facilities, where the proton energies would be of strong interest for material science, medical applications and laboratory astrophysics.

## Methods

### Nanowire Production

The Cu nanowire arrays were fabricated by the electrochemical deposition method, based on the methodology of Mondal et al.^44^ which is adapted from Gao et al.^65^. The through-hole Anodic Aluminum Oxide (AAO) membrane (pore size: 0.2 $\upmu$m, membrane thickness: 60 $\upmu$m, WHATMAN Anodisc) was applied as the template for the electrochemical deposition of Cu NWs. With a layer of gold (300 nm) sputtered on the one side, the AAO membrane served as the working cathode electrode in a conventional three-electrode cell for the electrochemical deposition. The graphite carbon and the saturated calomel electrode (SCE) were applied as the counter and the reference electrode, respectively. The electrolyte was a mixture of 0.2 M CuSO$_4$ and 0.1 M H$_3$BO$_3$. Experiments were carried out by using a potentiostat (Autolab) with the constant potential of -1.20 V (vs. SCE) at room temperature. The length of the Cu NWs can be controlled between 0 and 20 $\upmu$m by adjusting the deposition time during the synthesis. The nanowire diameter and the gap size were dictated by the template itself, which were around 200 nm for both and further confirmed by the Scanning Electron Microscope (SEM) characterization. For the SEM characterization, the as-prepared Cu nanowires embedded in the template were first immersed in a 1 M NaOH solution for 20 min to dissolve the AAO membrane. Then, they were rinsed in distilled water several times and let dry for 24 h prior to the shots. Special multi-target holders have been developed to perform multiple laser shots on one template. Prior to the laser shots, the AAO templates were dissolved inside the multi-target holder using the aforementioned methodology.

### PIC simulations

The PIC simulations involve a Gaussian p-polarized laser pulse incident at 20$^\circ$ with respect to target-normal, at a wavelength of $\lambda _0 = 800$ nm, a pulse duration of $\tau _L = 35$ fs at Full-Width Half-Maximum (FWHM), focused down to a Gaussian focal spot size of $w_\text {FWHM} = 3\,\upmu$m on the target at an intensity of $6\times 10^{19}$ W/cm$^2$, leading to $a_0 = 5.3$ where $a_0$ is the normalized amplitude of the vector potential. The simulation grid uses $\Delta x = \Delta y = 20$ nm, $\Delta t = \Delta x/c = 66$ as and runs over 800 fs. The box size is of $4080\times 4000$ cells ($102 \lambda _0 \times 100 \lambda _0$) in the transverse and longitudinal axes respectively. Copper nanowires were placed on a 300 nm thick gold foil, both with electron densities of $100 n_\text {c}$, where $n_\text {c}$ is the critical density at $\lambda _0 = 800$ nm. A 20 nm proton layer was placed at the back of the target with a density of $15 n_\text {c}$. Copper and gold ion species were initiated at the $3+$ ionization level with field and collisional ionizations enabled. We used 30 macroparticles per cell for electrons and the corresponding numbers for ion species to ensure charge neutrality in each cell at the initial state of the simulation. The investigated geometrical parameters were lengths of *l* = 0, 0.5, 1, 2, 5 and 10 $\upmu$m, diameters of *d* = 0, 100, 200, 400, 800 and 1600 nm, as well as gaps of *g* = 0, 100, 200, 400, 800 and 1600 nm. Only one NW parameter was varied at a time around the nominal NW parameters of $d=200$ nm, $g=200$ nm and $l=1\,\upmu$m. Each simulation runs 16 h on 600 cores.
